# Lung cancer risk in never-smokers: a population-based case-control study of epidemiologic risk factors

**DOI:** 10.1186/1471-2407-10-285

**Published:** 2010-06-14

**Authors:** Darren R Brenner, Rayjean J Hung, Ming-Sound Tsao, Frances A Shepherd, Michael R Johnston, Steven Narod, Warren Rubenstein, John R McLaughlin

**Affiliations:** 1 Prosserman Centre for Health Research, Samuel Lunenfeld Research Institute, 60 Murray St., Toronto, M5T 3L9, Canada; 2 Dalla Lana School of Public Health, University of Toronto, 155 College St., Toronto, Ontario, M5T 3M7, Canada; 3 Faculty of Medicine, University of Toronto, 1 King's College Circle, Toronto, Ontatrio, M5S 1A8, Canada; 4 Princess Margaret Hospital, 610 University Ave., Toronto, Ontario, M5G 2M9, Canada; 5 Women's College Health Research Institute, 790 Bay Street, Toronto, Ontario, M5G 1N8, Canada; 6 Mount Sinai Hospital Family Medicine Clinic, 60 Murray St., Toronto, M5T 3L9, Canada; 7 Population Studies and Surveillance, Cancer Care Ontario, 620 University Ave. Toronto Ontario, M5G 2L7, Canada

## Abstract

**Background:**

We conducted a case-control study in the greater Toronto area to evaluate potential lung cancer risk factors including environmental tobacco smoke (ETS) exposure, family history of cancer, indoor air pollution, workplace exposures and history of previous respiratory diseases with special consideration given to never smokers.

**Methods:**

445 cases (35% of which were never smokers oversampled by design) between the ages of 20-84 were identified through four major tertiary care hospitals in metropolitan Toronto between 1997 and 2002 and were frequency matched on sex and  ethnicity with 425 population controls and 523 hospital controls. Unconditional logistic regression models were used to estimate adjusted odds ratios (OR) and 95% confidence intervals (CI) for the associations between exposures and lung cancer risk.

**Results:**

Any previous exposure to occupational exposures (OR total population 1.6, 95% CI 1.4-2.1, OR never smokers 2.1, 95% CI 1.3-3.3), a previous diagnosis of emphysema in the total population (OR 4.8, 95% CI 2.0-11.1) or a first degree family member with a previous cancer diagnosis before age 50 among never smokers (OR 1.8, 95% CI 1.0-3.2) were associated with increased lung cancer risk.

**Conclusions:**

Occupational exposures and family history of cancer with young onset were important risk factors among never smokers.

## Background

Lung cancer is the most common type of cancer and the leading cause of cancer death in Canada with approximately 23,400 new cases (14.3 percent of all new cancers among males, 13.1 percent among females) and 20,500 deaths annually [1]. Although active tobacco smoking has been well established as the major cause of lung cancer [2-5], the etiology among never smokers beyond ETS exposure [6] remains to be elucidated and is of great public health importance [7,8]. The influences of indoor air pollution, workplace exposures and previous history of respiratory disease on lung cancer development among never smokers require additional investigation.

To further understand the etiology of lung cancer, with special consideration given to never smokers, we conducted a case-control study in the greater Toronto area with oversampling among never smokers. The objective of this study was to evaluate potential lung cancer risk factors including ETS exposure, family history of cancer, indoor air pollution, workplace exposures and history of previous respiratory diseases.

## Methods

### Study population and data collection

The case series consisted of incident cases of cancer of the trachea, bronchus or lung diagnosed among men and women between the ages of 20 and 84. Cases were identified between 1997 and 2002 through four major tertiary care hospitals in metropolitan Toronto that have the largest lung cancer services for surgical and medical oncology, including the two centres that see all patients who require radiotherapy. Diagnoses were histologically confirmed by a pulmonary pathologist following classification according to the ICD for oncology-3 [9]. As one of the main objectives of the study was to study lung cancer etiology other than from tobacco exposure, among never smokers, we therefore over sampled never smoking lung cancer patients, leading to 35% of the total cases being never smokers. A total of 445 eligible cases and 948 controls were recruited into the study for whom consent was obtained. Controls were residents of metropolitan Toronto who did not have cancer at the time of recruitment. Population-based controls were randomly sampled from property tax assessment files (n = 425). Hospital-based controls were sampled from patients seen in the Mount Sinai Hospital Family Medicine Clinic (n = 523), which is a non-specialty, family medicine practice situated within the hospital where recruitment into the study was conducted independent of reason for visit to the clinic. Controls were frequency matched with cases on sex and ethnicity. Participation rates were similar between cases (62%, 445 of 716 total eligible, 116 refused participation, whereas the remaining patients died before study entry and/or complete data collection was possible) and population controls, (60%, 425 of total 718 eligible) and slightly higher among hospital controls (84%, 523 of 621 total eligible). Informed consent was obtained from all participants and approvals were obtained from the Research Ethics Board.

### Exposure Information

Participants' lifetime information concerning tobacco consumption, exposure to ETS exposure, air pollution from heating, workplace exposures to potential lung carcinogens, family history of cancer and health history were collected through a detailed questionnaire administered via interview either in person or over the telephone. 'Never smokers' were defined as those who had not smoked more than 100 cigarettes in their lifetime. 'Former smokers' were smokers who had stopped smoking for at least two years at the date of the interview. Cumulative tobacco exposure was estimated in pack-years, where a pack is 20 cigarette equivalents.

Environmental tobacco smoke (ETS) exposure was categorized as having been exposed to second hand smoke either during childhood, as an adult or at work, with duration in years categorized to examine dose-response relationships. Indoor air pollution from heating was collected for oil, gas, coal & wood sources, with the duration of each exposure recorded. A measure of solid fuels for heating (coal and wood) was also created to examine the potential for differential effects of heating sources with particulate matter emission. Workplace exposures to potential lung carcinogens including asbestos, paints and/or solvents, welding equipment, pesticides, grain elevator dust, wood dust and smoke, soot or exhaust (not from tobacco) were dichotomized as exposed or unexposed. Family history was classified as the number of first degree relatives with any cancer, lung cancer, or aerodigestive tract cancers with distinction by relative types.

### Statistical Analysis

Differences in demographics between cases and controls as well as between control types were evaluated using χ^2 ^tests and t-tests. Multivariate unconditional logistic regression models were used to obtain odds ratio (OR) estimates and 95% confidence intervals (CI) for the associations between exposures and lung cancer risk, adjusted for cumulative tobacco exposures (pack-years), age (years), gender, education and ethnicity. Given that cases were sampled based on smoking status, all analyses were adjusted for smoking and the focus of this investigation is on factors other than tobacco. Indicator variables were created for all categorical variables in analyses. We stratified analyses by years of exposure and age of onset when applicable in an attempt to determine the temporality of potential exposure-disease associations. Analyses were conducted using SAS Version 9.1 (SAS V9.1; SAS Institute Inc, Cary NC, USA).

We also applied the Spitz (2007) [10] and Liverpool Lung Project (2008) [11] lung cancer risk models to evaluate the predictive ability of their models within our population. We stratified our population by smoking status to examine the area under the curve and Hosmer Lemeshow goodness-of-fit statistics [12] within the subgroups. Previous history of hay fever and dust exposure were not available in our study and were thus not included as part of the Spitz model.

## Results

Table 1 shows the frequency distribution of demographic variables and smoking for cases and controls. Controls were younger and more educated. There was a higher percentage of never smokers among controls than cases. Among cases, adenocarcinoma was the most common histological subtype with a higher proportion of adenocarcinomas among never smoking cases. Never smoking cases also consisted of a higher proportion of females and Asians and were on average younger at diagnosis (p < .0001). When examining demographic differences between population and hospital based controls, controls varied across gender, education, age groups and smoking groups. However, when examining age and pack-years smoking as included in regression models, we observed no significant differences across control types.

**Table 1:** Demographic characteristics of lung cancer patients and controls in a population based case-control study, Greater Toronto Area, Ontario, 1997- 2002

	Case, *n*(%)			Control, *n*(%)			
	**Smokers**	**Never smokers**	**Total**	**Smokers**	**Never smokers**	**Total**	**p value^a^**
Total	289	156	445	482	466	948	
							
Gender							
Male	163 (56)	46 (30)	209 (47)	241 (50)	145 (31)	386 (41)	p = 0.03
Female	126 (44)	110 (70)	236 (53)	241 (50)	321 (69)	562 (59)	
Age							
< 35	1 (< 1)	10 (6)	11 (3)	58 (12)	86 (18)	144 (15)	
35-45	9 (3)	21 (13)	30 (7)	77 (16)	89 (19)	166 (180	
45-55	34 (12)	27 (17)	61 (14)	88 (18)	85 (18)	173 (18)	
55-65	63 (22)	28 (18)	91 (20)	101 (21)	88 (19)	189 (20)	
65-75	131 (45)	57 (37)	188 (42)	100 (21)	71 (15)	170 (18)	
> 75	51 (18)	13 (8)	64 (14)	58 (12)	48 (10)	106 (11)	
							
Age, Mean ± SD	66 ± 10	59 ± 13	64 ± 12	56 ± 16	53 ± 17	54 ± 16	< .0001
							
Ethnicity							
White	255 (88)	97 (62)	352 (79)	429 (89)	346 (74)	775 (82)	p = 0.07
Asian	21 (7)	48 (31)	69 (16)	27 (6)	78 (17)	105 (11)	
Other	13 (5)	11 (7)	24 (5)	26 (5)	42 (9)	68 (7)	
							
Education							
< 8 years	97 (34)	36 (23)	137 (31)	55 (11)	68 (15)	140 (15)	< .0001
8-11 years	137 (47)	52 (33)	189 (42)	212 (44)	187 (40)	399 (42)	
≥12 years	55 (19)	64 (41)	119 (27)	205 (43)	204 (44)	409 (43)	
							
All types of smoking combined							
Never		156	156 (35)		466	466 (49)	< .0001
Former (> 2 yrs. Since quitting)	159 (55)		159 (36)	319 (66)		319 (34)	
Current	130 (45)		130 (29)	163 (34)		163 (17)	
							
Pack-years^b^, Mean ± SD	45 ± 35			25 ± 27			< .0001
							
Histology							
Adenocarcinoma	80 (28)	76 (49)	156 (35)				
Squamous cell carcinoma	68 (24)	9 (6)	77 (17)				
Small-cell carcinoma	28 (10)	4 (3)	32 (7)				
Large cell carcinoma	21 (7)	6 (4)	27 (6)				
Others/Mixed	46 (16)	26 (17)	72 (16)				
Not classified/clinical diagnosis	46 (16)	35 (22)	81 (18)				

^a ^p-values from χ^2 ^test or t-test across cases and controls

^b ^Pack-years among ever and current smokers

Cases and controls did not vary significantly in the total hours exposed to ETS during childhood or adulthood at home (data not shown). Among never smokers in our population, we observed no association between either exposure to ETS at home or at the workplace and lung cancer risk (Table 2). In general, the effect estimates for ETS exposure were similar between the total population and only among never smokers. In terms of indoor air pollution, we did not observe a significant association between heating source (coal and/or wood) and lung cancer risk among never smokers (OR 1.5, 95% CI 0.1-2.8).

**Table 2:** The association between ETS and risk of lung cancer among never smokers in a population based case-control study, Greater Toronto Area, Ontario, 1997-2002

	Total Population n = 1393				Never smokers n = 622			
	**Case, *n***	**Control, *n***	**OR^a^**	**95%CI**	**Case, *n***	**Control, *n***	**OR^b^**	**95%CI**
	445	948			156	466		
ETS Exposure								
None	29	88	Ref^c^		23	68	Ref^d^	
At home Adult and/or Child	375	772	1.2	0.7-1.9	109	341	1.1	0.6-1.9
Childhood	333	672	1.1	0.7-1.9	93	298	1.0	0.6-1.8
Adulthood	226	439	1.0	0.6-1.7	50	247	1.0	0.5-2.0
								
At work	259	463	1.3	0.9-1.9	69	179	1.2	0.7-2.1
< 10 years	73	194	1.1	0.8-1.6	32	90	1.3	0.8-2.2
> 10 years	176	261	1.2	0.9-1.6	37	86	1.2	0.7-2.0
								
At both home and work	226	405	1.4	0.9-2.2	50	142	1.2	0.7-2.1

^a^OR is adjusted for pack-years of smoking, age, sex, education and ethnicity

^b^OR is adjusted for age, sex, education and ethnicity

^c^Reference group consists of all participants with no ETS exposure

^d^Reference group consists of never-smokers with no ETS exposure

The association between occupational exposures to asbestos, solvents, paints or thinners, welding equipment, pesticides, grain elevator dust, wood dust and smoke, soot or exhaust (from sources other than tobacco) and lung cancer risk is shown in Table 3. Occupational exposure to any of the putative lung carcinogens was associated with lung cancer risk in the total population (OR 1.6, 95% CI 1.4-2.1). Among never smokers, the odds ratio for exposure to any of the putative carcinogens was 2.1 (95% CI 1.3- 3.3). Specifically among never smokers, exposure to solvents, paints or thinners conferred an OR of 2.8 (95% CI 1.6-5.0), while exposure to welding equipment conferred an OR of 3.4 (95% CI 1.1-10.4) and exposure to smoke, soot or exhaust (other than tobacco) conferred an OR of 2.8 (95% CI 1.4-5.3). We did not observe significant associations for exposures to asbestos, pesticides, grain elevator dust, and wood dust among never smokers in our study.

**Table 3:** The association between workplace exposures and risk of lung cancer in a population based case-control study, Greater Toronto Area, Ontario, 1997-2002

	Total Population n = 1393				Never smokers n = 622			
	**Case, *n***	**Control, *n***	**OR^a^**	**95%CI**	**Case, *n***	**Control, *n***	**OR^b^**	**95%CI**
	445	948			156	466		
								
No previous exposures	261	691		Ref^c^	104	371		Ref^d^
								
Any occupational exposure	191	275	1.6	1.4-2.1	52	95	2.1	1.3-3.3
								
Ever worked with/been exposed to:								
								
Asbestos	31	51	1.1	0.6-2.0	5	16	1.0	0.3-3.0
								
Solvents, paints or thinners	107	152	1.6	1.2-2.3	33	54	2.8	1.6-5.0
								
Welding equipment	33	43	1.7	1.0-3.0	7	11	3.4	1.1-10.4
								
Pesticides	21	24	1.6	0.8-3.1	3	6	1.1	0.2-5.3
								
Grain elevator dust	12	19	1.1	0.5-2.4	3	7	1.1	0.3-4.6
								
Wood dust	47	80	1.5	1.0-2.4	11	26	1.8	0.8-4.2
								
smoke-soot or exhaust	75	99	1.7	1.2-2.5	21	33	2.8	1.4-5.3
other than tobacco								

^a^OR is adjusted for pack-years of smoking, age, sex, education and ethnicity

^b^OR is adjusted for age, sex, education and ethnicity

^c^Reference group consists of all participants with no previous workplace exposures

^d^Reference group consists of never smokers with no previous workplace exposures

With regard to previous medical history of respiratory conditions, we observed a significant increase in lung cancer risk associated with a previous diagnosis of emphysema among the total population (OR 4.8, 95 % CI 2.0-11.1) (Table 4). When stratified by age at onset, those with age of onset of emphysema greater than 50 years old had a significant increase in lung cancer risk (OR 4.2, 95% CI 1.0-10.9), whereas among those less than 50 years of age, the risk was not significant (OR 4.0, 95% CI 0.6-27.5) (Data not shown). None of the other previous conditions we investigated (asthma, chronic bronchitis, pneumonia or tuberculosis) were associated with increased risk. In a model including all previous lung disease, the effects of emphysema maintained significance.

**Table 4:** The association between previous medical history and risk of lung cancer in a population based case-control study, Greater Toronto Area, Ontario, 1997-2002

	Total Population n = 1393				Never smokers n = 622			
	**Case, *n***	**Control, *n***	**OR^a^**	**95%CI**	**Case, *n***	**Control, *n***	**OR^b^**	**95%CI**
	445	948			156	466		
								
Emphysema								
Never	414	940	1.0	Ref	154	465	1.0	Ref
Ever	31	8	4.8	2.0-11.1	2	1	3.1	0.3-35.9
								
Chronic Bronchitis								
Never	424	899	1.0	Ref	153	440	1.0	Ref
Ever	21	49	0.9	0.5-1.7	3	26	0.4	0.1-1.5
								
Asthma								
Never	388	835	1.0	Ref	140	408	1.0	Ref
Ever	57	113	1.2	0.8-1.8	16	58	1.0	0.5-1.9
								
Pneumonia								
Never	434	913	1.0	Ref	151	456	1.0	Ref
Ever	11	35	0.6	0.3-1.2	5	10	1.9	0.6-6.2
								
Tuberculosis								
Never	439	943	1.0	Ref	153	463	1.0	Ref
Ever	6	5	2.6	0.7-9.2	3	3	2.2	0.4-12.5
								
Other respiratory illness								
Never	385	823	1.0	Ref	139	403	1.0	Ref
Ever	60	125	1.1	0.8-1.6	17	63	0.9	0.5-1.7

^a^OR is adjusted for pack-years of smoking, age, sex, education and ethnicity

^b^OR is adjusted for age, sex, education and ethnicity

We did not observe a significant association between family history of cancer and lung cancer risk, except among those with affected relatives with young onset (< 50 years of age) cases (Table 5). Among never smokers having a relative with young onset cancer was associated with a significant increase in risk (OR 1.8, 95% CI 1.0-3.2, (p = 0.04)). An increasing number of first degree relatives with a previous history of any cancer suggested an increase in risk for having 2 or more family members. Trend statistics for increasing number of first degree relatives with cancer were, however, not significant (p-trend = 0.2). No association was detected when data were analyzed by the type of affected relatives (data not shown).

**Table 5:** The association between family history of previous cancer and risk of lung cancer in a population based case-control study, Greater Toronto Area, Ontario, 1997-2002

	Total Population n = 1393				Never smokers n = 622			
	**Case, *n***	**Control, *n***	**OR^a^**	**95%CI**	**Case, *n***	**Control, *n***	**OR^b^**	**95%CI**
	445	948			156	466		
								
No family history of any cancer	246	563	1.0	Ref^c^	95	293	1.0	Ref^d^
								
Positive family history of any cancer								
1	141	292	1.1	0.8-1.4	42	134	0.9	0.6-1.4
2 or more	58	93	1.3	0.9-1.9	19	39	1.4	0.8-2.8
Affected relatives age at onset < 50	82	126	1.3	0.9-1.9	28	48	1.8	1.0-3.2
								
Positive family history of aero-digestive cancer	45	78	1.2	0.8-1.8	11	34	0.9	0.4-1.9
								
Positive family history of lung cancer	30	63	1.0	0.6-1.7	8	26	0.9	0.4-2.1

^a^Adjusted for pack-years smoking, age, sex, education and ethnicity

^b^Adjusted for age, sex, education and ethnicity

^c^Reference group consists of all participants with no family history of cancer

^d^Reference group consists of never smokers with no family history of cancer

Applying the Spitz risk model indicated that there was only modest predictive ability among never smokers in our population (Area under the Curve (AUC) 0.525). Among smokers (current and former), however, the Spitz model was shown to have better predictive power, (AUC former smokers = 0.716, current smokers = 0.780), despite our study not possessing data for hay fever or dust exposure. The Liverpool Lung Project risk model provided similar outcomes in prediction, identifying cases in the total population well (AUC 0.788) with lower statistics when applied to only never smokers in the population (AUC 0.721).

## Discussion

In this study we investigated the impact of several factors on lung cancer risk overall as well as specifically among never smokers. The most important risk factors we observed among never smokers were exposure to potential occupational carcinogens, family history of cancer with young onset and previous history of respiratory diseases among the total population.

In our examination of the effects of several occupational exposures among never smokers in the greater Toronto area we found several significant potential sources of increased risk including exposure to solvents, paints or thinners, welding equipment and smoke, soot or exhaust (from sources other than tobacco). This information is important as data concerning occupational exposures and lung cancer among never smokers are still lacking in the literature [13].

Our results support the concept that exposure to exhaust fumes and or soot/smoke (from non-tobacco sources) is a source of carcinogenic exposure. A previous meta-analysis suggested that when adjusted for smoking, heavy diesel exhaust exposure was associated with an increased risk (OR 1.4, 95% CI 1.3-1.6) [14], and a recent study examining the effects in a similar Canadian population, was also suggestive of increased risk (OR 1.6, 95% CI 0.9-2.8) [15]. With regards to soot and exhaust exposure, these substances contain benzo[a]pyrene, a known carcinogen, and has been consistently shown to increase risk [16,17]. We observed an increased risk associated with exposure to paints, thinners and solvents, which was in agreement with previous studies [18-22]. When ingested, these substances can affect the pleural membranes, causing scarring and or mutations, thus increasing the potential for carcinogenesis [23]. Similarly, exposure to welding equipment was associated with increased risk as observed in a meta-analysis of welding and lung cancer [24]. Wood dust is a known carcinogen associated with the development of cancers of the respiratory tract [25-27]. In this study the estimate for wood dust exposure was suggestive of increased risk among never smokers. While these observations require replication, they are consistent with the overall patterns seen for wood dust, with the potential implication that workplace exposures should be controlled and monitored. Asbestos exposure has been previously shown to have an effect on lung cancer risk [28,29]; however, no association between lung cancer and asbestos was seen here among never smokers, contrary to previously published results [30]. The discrepancies with the previous studies may be due to an attenuation of the risk estimate as a result of the simple dichotomy used to indicate asbestos exposure which may not distinguish between actual or potential exposure among the small number of individuals reporting exposure in this non-occupational cohort. Overall, these observations provide support for efforts to control, monitor and reduce exposures to potentially hazardous workplace exposures, which in this study are shown to be associated with lung cancer, even among never smokers.

Our findings are consistent with the evidence suggesting that a previous history of acquired respiratory conditions is a risk factor for lung cancer [31-41]. Chronic inflammation and airway obstruction may predispose individuals to various types of cancer as the damage created by acquired pulmonary diseases such as chronic obstructive pulmonary disease (COPD) may be involved in cancer development [42-47]. Proposed biological mechanisms include enhanced effects of carcinogenic exposures in the presence of chronic inflammation or a compromised immune response [48,49], as well as the possibility of lung cancer evolving directly from the scar lesions created by non-malignant conditions [50,51]. Although the analyses performed here accounted for active smoking, it is still possible that the relationship between acquired respiratory disease and lung cancer is partially explained by residual confounding from tobacco [52]. In addition, due to the relatively small numbers further investigations among never smokers is still warranted. Further elucidation and characterization of the genetic variants associated with inflammation of the lungs may also help to clarify the role of acquired respiratory conditions in the etiology of lung cancer.

ETS exposure was not found to significantly increase risk among never smokers in this study, however, several potential explanations are possible. ETS exposure either as a child or an adult in the home or the workplace has been evaluated in numerous studies [53]. The results, however, have been inconsistent as to the significance and magnitude of the effects among never smokers. When estimates were pooled in a meta-analysis of 34 case-control studies of non-smokers, a pooled relative risk of 1.2 (95% CI 1.1-1.4) was observed, although only seven out of 34 studies reporting significantly elevated risk [6]. It was suggested that the inconsistency in the significance of findings across studies could be due to issues of sample size, measurement error, recall bias and confounding [54]. Despite our efforts to minimize misclassification bias by collecting data on involuntary tobacco smoke exposure data for home, work and other exposure locations during both childhood and adulthood, the possibility of these issues cannot be excluded. The main limitation in our study is the lack of power to detect a modest effect. Non-differential misclassification of the dichotomous exposures may also lead to a bias toward to null. We combined hospital and population based controls in an attempt to increase our sample size and in turn the ability to detect significant associations. In order to address any issues created by this pooling we investigated effect estimates among only population based controls. Effect estimates were of a similar magnitude and no significant associations were observed among population based controls that were not observed among the total population.

Another limitation of this study is its dependence on self-reported exposures. Previous history of respiratory disease was self-reported as access to patient medical records was not available for validation, and similarly, validation of occupation was not possible due to a lack of occupational records. Even so, this study provides risk estimates for a relatively large group of never smoking lung cancer cases in a population-based study, and thus yields findings that are of increasing relevance given recent changes in tobacco use in the population. The detailed risk factor information concerning indoor air pollution and family history collected from patients following diagnosis, as well as similar participation rates among cases and controls are additional strengths of the study.

When applying previously specified risk prediction models to our population, both models were able to adequately predict outcomes among smokers, however, both models had substantially less predictive ability among never smokers. This indicates that previously identified risk prediction models have little utility among never smokers and that additional determinants of increased risk or susceptibility must still be identified among this group. Identification of these new factors among never smokers has been difficult due to the small numbers of never smoking cases in studies to date. With the development of large-scale collaborations and consortia [55], it will become possible for much more detailed risk models to be evaluated among larger populations of never smokers, leading ultimately to improved risk prediction and understanding of lung cancer etiology among never smokers.

This study mainly assessed environmental risk factors for the development of lung cancer in never smokers. It is now clear that the molecular pathogenesis of lung cancer in smokers and non-smokers is different, with a higher proportion of adenocarincoma observed among never smoking cases. Recent studies have demonstrated that activating mutations in the *EGFR *tyrosine kinase domain occur much more frequently in lung cancers in non and never smoking patients. Furthermore, these mutations are found significantly more often in adenocarcinomas, women, and individuals of Asian origin where the mutation rate can reach 60% in patients with these characteristics [56]. These characteristics were all significantly higher in our never smoking subset. Unfortunately, we do not have adequate tissue samples to assess mutation status in our cases, but studies of the interaction between *EGFR *mutations and environmental factors deserve further investigation.

## Conclusion

In conclusion, occupational exposures displayed the strongest associations with increased lung cancer risk among never smokers in this study. Further understanding of the role of these factors in lung cancer etiology may ultimately lead to improved lung cancer prevention strategies for the whole population.

## Competing interests

The authors declare that they have no competing interests.

## Authors' contributions

DB carried out the analysis and drafted the manuscript. RH assisted in drafting of the manuscript and provided critical revision. MT aided in the design of the study and data collection. FS aided in the design of the study and data collection and provided critical revision of the manuscript. MJ aided in the design of the study and data collection. SN aided in the design of the study and data collection. WR aided in the design of the study and data collection. JM led the study design and data collection and coordinated the drafting of the manuscript while providing critical revision. All authors read and approved the final manuscript.

## Pre-publication history

The pre-publication history for this paper can be accessed here:

http://www.biomedcentral.com/1471-2407/10/285/prepub

## References

[B1] Canadian Cancer Society's Steering Committee Canadian Cancer Statistics 2009 2009 Toronto: Canadian Cancer Society

[B2] Daff ME Doll R Kennaway EL Cancer of the lung in relation to tobacco Br J Cancer 1951 5 1 1 20 14839198 10.1038/bjc.1951.1 PMC2007759

[B3] Doll R Peto R Wheatley K Gray R Sutherland I Mortality in relation to smoking: 40 years' observations on male British doctors Bmj 1994 309 6959 901 911 7755693 10.1136/bmj.309.6959.901 PMC2541142

[B4] Tobacco Smoke and Involuntary Smoking 2004 83 Lyon, France: IARC Press PMC4781536 15285078

[B5] Baron J Rohan TE Schottenfeld D, Fraumeni JF Tobacco Cancer epidemiology and Prevention 1996 2 New York, NY: Oxford University Press

[B6] Hackshaw AK Law MR Wald NJ The accumulated evidence on lung cancer and environmental tobacco smoke Bmj 1997 315 7114 980 988 9365295 10.1136/bmj.315.7114.980 PMC2127653

[B7] Subramanian J Govindan R Lung cancer in never smokers: a review J Clin Oncol 2007 25 5 561 570 10.1200/JCO.2006.06.8015 17290066

[B8] Sun S Schiller JH Gazdar AF Lung cancer in never smokers--a different disease Nat Rev Cancer 2007 7 10 778 790 10.1038/nrc2190 17882278

[B9] World Health Organization International Classification of Diseases for Oncology - 3 2000 Geneva, WHO

[B10] Spitz MR Hong WK Amos CI Wu X Schabath MB Dong Q Shete S Etzel CJ A risk model for prediction of lung cancer J Natl Cancer Inst 2007 99 9 715 726 10.1093/jnci/djk153 17470739

[B11] Cassidy A Myles JP van Tongeren M Page RD Liloglou T Duffy SW Field JK The LLP risk model: an individual risk prediction model for lung cancer Br J Cancer 2008 98 2 270 276 10.1038/sj.bjc.6604158 18087271 PMC2361453

[B12] Lemeshow S Hosmer DW Jr A review of goodness of fit statistics for use in the development of logistic regression models Am J Epidemiol 1982 115 1 92 106 7055134 10.1093/oxfordjournals.aje.a113284

[B13] Neuberger JS Field RW Occupation and lung cancer in nonsmokers Rev Environ Health 2003 18 4 251 267 15025189 10.1515/reveh.2003.18.4.251

[B14] Lipsett M Campleman S Occupational exposure to diesel exhaust and lung cancer: a meta-analysis Am J Public Health 1999 89 7 1009 1017 10.2105/AJPH.89.7.1009 10394308 PMC1508841

[B15] Parent ME Rousseau MC Boffetta P Cohen A Siemiatycki J Exposure to diesel and gasoline engine emissions and the risk of lung cancer Am J Epidemiol 2007 165 1 53 62 10.1093/aje/kwj343 17062632

[B16] Lloyd JW Long-term mortality study of steelworkers. V. Respiratory cancer in coke plant workers J Occup Med 1971 13 2 53 68 10.1097/00043764-197102000-00001 5546197

[B17] Doll R Fisher RE Gammon EJ Gunn W Hughes GO Tyrer FH Wilson W Mortality of Gasworkers with Special Reference to Cancers of the Lung and Bladder, Chronic Bronchitis, and Pneumoconiosis Br J Ind Med 1965 22 1 12 14261702 10.1136/oem.22.1.1 PMC1008209

[B18] Zeka A Mannetje A Zaridze D Szeszenia-Dabrowska N Rudnai P Lissowska J Fabianova E Mates D Bencko V Navratilova M Lung cancer and occupation in nonsmokers: a multicenter case-control study in Europe Epidemiology 2006 17 6 615 623 10.1097/01.ede.0000239582.92495.b5 17068414

[B19] Stockwell HG Matanoski GM A case-control study of lung cancer in painters J Occup Med 1985 27 2 125 126 3872357

[B20] Lerchen ML Wiggins CL Samet JM Lung cancer and occupation in New Mexico J Natl Cancer Inst 1987 79 4 639 645 3477658

[B21] Muscat JE Stellman SD Richie JP Jr Wynder EL Lung cancer risk and workplace exposures in black men and women Environ Res 1998 76 2 78 84 10.1006/enrs.1997.3787 9515062

[B22] Chen R Seaton A A meta-analysis of painting exposure and cancer mortality Cancer Detect Prev 1998 22 6 533 539 10.1046/j.1525-1500.1998.00A47.x 9824376

[B23] IARC Monographs programme on the evaluation of the carcinogenic risk of chemicals to humans. Preamble IARC Monogr Eval Carcinog Risk Chem Hum 1986 39 13 32 3465680

[B24] Ambroise D Wild P Moulin JJ Update of a meta-analysis on lung cancer and welding Scand J Work Environ Health 2006 32 1 22 31 16539169 10.5271/sjweh.973

[B25] Jayaprakash V Natarajan KK Moysich KB Rigual NR Ramnath N Natarajan N Reid ME Wood Dust Exposure and the Risk of Upper Aero-Digestive and Respiratory Cancers in Males Occup Environ Med 2008 65 10 647 54 10.1136/oem.2007.036210 18182588

[B26] Baran S Teul I Wood dust: an occupational hazard which increases the risk of respiratory disease J Physiol Pharmacol 2007 58 Suppl 5 Pt 1 43 50 18204114

[B27] Barcenas CH Delclos GL El-Zein R Tortolero-Luna G Whitehead LW Spitz MR Wood dust exposure and the association with lung cancer risk Am J Ind Med 2005 47 4 349 357 10.1002/ajim.20137 15776474

[B28] Hillerdal G Henderson DW Asbestos, asbestosis, pleural plaques and lung cancer Scand J Work Environ Health 1997 23 2 93 103 9167232 10.5271/sjweh.186

[B29] Gustavsson P Nyberg F Pershagen G Scheele P Jakobsson R Plato N Low-dose exposure to asbestos and lung cancer: dose-response relations and interaction with smoking in a population-based case-referent study in Stockholm, Sweden Am J Epidemiol 2002 155 11 1016 1022 10.1093/aje/155.11.1016 12034580

[B30] Neuberger JS Mahnken JD Mayo MS Field RW Risk factors for lung cancer in Iowa women: implications for prevention Cancer Detect Prev 2006 30 2 158 167 10.1016/j.cdp.2006.03.001 16581199 PMC1876736

[B31] Skillrud DM Offord KP Miller RD Higher risk of lung cancer in chronic obstructive pulmonary disease. A prospective, matched, controlled study Ann Intern Med 1986 105 4 503 507 3752756 10.7326/0003-4819-105-4-503

[B32] Mayne ST Buenconsejo J Janerich DT Previous lung disease and risk of lung cancer among men and women nonsmokers Am J Epidemiol 1999 149 1 13 20 9883789 10.1093/oxfordjournals.aje.a009722

[B33] Samet JM Humble CG Pathak DR Personal and family history of respiratory disease and lung cancer risk Am Rev Respir Dis 1986 134 3 466 470 3752703 10.1164/arrd.1986.134.3.466

[B34] Alavanja MC Brownson RC Boice JD Jr Hock E Preexisting lung disease and lung cancer among nonsmoking women Am J Epidemiol 1992 136 6 623 632 1442729 10.1093/oxfordjournals.aje.a116542

[B35] Brenner AV Wang Z Kleinerman RA Wang L Zhang S Metayer C Chen K Lei S Cui H Lubin JH Previous pulmonary diseases and risk of lung cancer in Gansu Province, China Int J Epidemiol 2001 30 1 118 124 10.1093/ije/30.1.118 11171871

[B36] Wu AH Fontham ET Reynolds P Greenberg RS Buffler P Liff J Boyd P Henderson BE Correa P Previous lung disease and risk of lung cancer among lifetime nonsmoking women in the United States Am J Epidemiol 1995 141 11 1023 1032 7771438 10.1093/oxfordjournals.aje.a117366

[B37] Talbot-Smith A Fritschi L Divitini ML Mallon DF Knuiman MW Allergy, atopy, and cancer: a prospective study of the 1981 Busselton cohort Am J Epidemiol 2003 157 7 606 612 10.1093/aje/kwg020 12672680

[B38] Osann KE Lowery JT Schell MJ Small cell lung cancer in women: risk associated with smoking, prior respiratory disease, and occupation Lung Cancer 2000 28 1 1 10 10.1016/S0169-5002(99)00106-3 10704703

[B39] Santillan AA Camargo CA Jr Colditz GA A meta-analysis of asthma and risk of lung cancer (United States) Cancer Causes Control 2003 14 4 327 334 10.1023/A:1023982402137 12846363

[B40] Boffett P Ye W Boman Nyren G Lung cancer risk in a population-based cohort of patients hospitalized for asthma in Sweden Eur Respir J 2002 19 1 127 133 10.1183/09031936.02.00245802 11843311

[B41] Ramanakumar AV Parent ME Menzies D Siemiatycki J Risk of lung cancer following nonmalignant respiratory conditions: evidence from two case-control studies in Montreal, Canada Lung Cancer 2006 53 1 5 12 10.1016/j.lungcan.2006.04.007 16733074

[B42] O'Byrne KJ Dalgleish AG Chronic immune activation and inflammation as the cause of malignancy Br J Cancer 2001 85 4 473 483 10.1054/bjoc.2001.1943 11506482 PMC2364095

[B43] Seow A Ng DP Choo S Eng P Poh WT Ming T Wang YT Joint effect of asthma/atopy and an IL-6 gene polymorphism on lung cancer risk among lifetime non-smoking Chinese women Carcinogenesis 2006 27 6 1240 1244 10.1093/carcin/bgi309 16344268

[B44] Bauer AK Malkinson AM Kleeberger SR Susceptibility to neoplastic and non-neoplastic pulmonary diseases in mice: genetic similarities Am J Physiol Lung Cell Mol Physiol 2004 287 4 L685 703 10.1152/ajplung.00223.2003 15355860

[B45] Schabath MB Delclos GL Martynowicz MM Greisinger AJ Lu C Wu X Spitz MR Opposing effects of emphysema, hay fever, and select genetic variants on lung cancer risk Am J Epidemiol 2005 161 5 412 422 10.1093/aje/kwi063 15718477

[B46] Suzuki K Ito Y Wakai K Kawado M Hashimoto S Seki N Ando M Nishino Y Kondo T Watanabe Y Serum heat shock protein 70 levels and lung cancer risk: a case-control study nested in a large cohort study Cancer Epidemiol Biomarkers Prev 2006 15 9 1733 1737 10.1158/1055-9965.EPI-06-0005 16985037

[B47] Yang P Wentzlaff KA Katzmann JA Marks RS Allen MS Lesnick TG Lindor NM Myers JL Wiegert E Midthun DE Alpha1-antitrypsin deficiency allele carriers among lung cancer patients Cancer Epidemiol Biomarkers Prev 1999 8 5 461 465 10350443

[B48] Ohshima H Bartsch H Chronic infections and inflammatory processes as cancer risk factors: possible role of nitric oxide in carcinogenesis Mutat Res 1994 305 2 253 264 7510036 10.1016/0027-5107(94)90245-3

[B49] Zheng W Blot WJ Liao ML Wang ZX Levin LI Zhao JJ Fraumeni JF Jr Gao YT Lung cancer and prior tuberculosis infection in Shanghai Br J Cancer 1987 56 4 501 504 2825752 10.1038/bjc.1987.233 PMC2001820

[B50] Madri JA Carter D Scar cancers of the lung: origin and significance Hum Pathol 1984 15 7 625 631 10.1016/S0046-8177(84)80286-5 6378757

[B51] Xie L Ugnat AM Morriss J Semenciw R Mao Y Histology-related variation in the treatment and survival of patients with lung carcinoma in Canada Lung Cancer 2003 42 2 127 139 10.1016/S0169-5002(03)00283-6 14568680

[B52] Boffetta P Ye W Boman Nyren G Lung cancer risk in a population-based cohort of patients hospitalized for asthma in Sweden Eur Respir J 2002 19 1 127 133 10.1183/09031936.02.00245802 11843311

[B53] Boffetta P Involuntary smoking and lung cancer Scand J Work Environ Health 2002 28 Suppl 2 30 40 12058801

[B54] Lee PN Difficulties in assessing the relationship between passive smoking and lung cancer Stat Methods Med Res 1998 7 2 137 163 10.1191/096228098669795194 9654639

[B55] Hung RJ Christiani DC Risch A Popanda O Haugen A Zienolddiny S Benhamou S Bouchardy C Lan Q Spitz MR International Lung Cancer Consortium: pooled analysis of sequence variants in DNA repair and cell cycle pathways Cancer Epidemiol Biomarkers Prev 2008 17 11 3081 3089 10.1158/1055-9965.EPI-08-0411 18990748 PMC2756735

[B56] Mok TS Wu YL Thongprasert S Yang CH Chu DT Saijo N Sunpaweravong P Han B Margono B Ichinose Y Gefitinib or carboplatin-paclitaxel in pulmonary adenocarcinoma N Engl J Med 2009 361 10 947 957 10.1056/NEJMoa0810699 19692680

